# Prioritizing the Effects of Emerging Contaminants on Estuarine Production under Global Warming Scenarios

**DOI:** 10.3390/toxics10020046

**Published:** 2022-01-20

**Authors:** Irene Martins, Joana Soares, Teresa Neuparth, Aldo F. Barreiro, Cândido Xavier, Carlos Antunes, Miguel M. Santos

**Affiliations:** 1 CIMAR/CIIMAR—Interdisciplinary Centre of Marine and Environmental Research, University of Porto, Av. General Norton de Matos S/N, 4450-208 Matosinhos, Portugal; jsoares@ciimar.up.pt (J.S.); tneuparth@ciimar.up.pt (T.N.); abarreiro@ciimar.up.pt (A.F.B.); cantunes@ciimar.up.pt (C.A.); 2 FCUP—Faculty of Sciences, University of Porto, Rua do Campo Alegre S/N, 4169-007 Porto, Portugal; candido.cacx@gmail.com; 3 Aquamuseu do Rio Minho, Parque do Castelinho, 4920-290 Vila Nova de Cerveira, Portugal

**Keywords:** AQUATOX, ecosystem model, standard regression coefficients, general linear model, endocrine disrupting chemicals, BPA, *Scrobicularia plana*, *Carcinus maenas*

## Abstract

Due to non-linear interactions, the effects of contaminant mixtures on aquatic ecosystems are difficult to assess, especially under temperature rise that will likely exacerbate the complexity of the responses. Yet, under the current climatic crisis, assessing the effects of water contaminants and temperature is paramount to understanding the biological impacts of mixtures of stressors on aquatic ecosystems. Here, we use an ecosystem model followed by global sensitivity analysis (GSA) to prioritize the effects of four single emerging contaminants (ECs) and their mixture, combined with two temperature rise scenarios, on the biomass production of a NE Atlantic estuary. Scenarios ran for 10 years with a time-step of 0.1 days. The results indicate that macroinvertebrate biomass was significantly explained by the effect of each single EC and by their mixture but not by temperature. Globally, the most adverse effects were induced by two ECs and by the mixture of the four ECs, although the sensitivity of macroinvertebrates to the tested scenarios differed. Overall, the present approach is useful to prioritize the effects of stressors and assess the sensitivity of the different trophic groups within food webs, which may be of relevance to support decision making linked to the sustainable management of estuaries and other aquatic systems.

## 1. Introduction

Aquatic ecosystems are under the influence of complex mixtures of contaminants resulting mostly from human activities [1]. River basins and estuaries in particular are largely affected by contaminant mixtures, as they receive numerous discharges from sewage treatment plants, agriculture, industry and urbanization along their catchment areas on their seaward transport [2,3,4]. Although baseline studies reporting that concentrations of metals, dioxins, furans [5] and emerging contaminants such as pharmaceuticals, personal care products and industrial compounds are abundant [6,7,8,9], works referring to the impacts of mixtures of contaminants in natural populations are scarce (but see [10,11]). This is due to the difficulties linked to disentangling the effects of environmental factors and those from contaminants, performing rigorous long-term experiments and the costs associated with time and money [12,13,14,15]. Nonetheless, categorizing interactions among factors and prioritizing stressors is important to guide decision making and to undertake cost-effective solutions regarding the mitigation and management of coastal systems [16]. Additionally, climate change-related oscillations will sum up this complexity as suggested by both experimental and numerical works [17,18,19].

Ecosystem models are tools that incorporate physical, biogeochemical and physiological processes acting on ecosystems, accounting for oscillations of different factors and projecting future variations [20]. Models of estuarine food webs have been used to understand the effects of different events occurring within the same estuary at different timing, disturbance events (eutrophication and an extreme flood) and the undertaking of mitigation measures on benthic food web properties [21]. The results indicate that distinct disturbances have different impacts on the food web structure and that due to direct and indirect interactions between the all-interacting processes, whole system approaches are required.

Furthermore, due to their integrative properties and dynamic nature, models can also provide evidence for which stressor is dominant in a system and unveil the type of interactions among different stressors [18,22].

However, there is still a substantial lack of multiple stressor and multi-pollutant models [1] capable of accounting for various pressures interactions and projecting the system variations in the long-term [15]. Nonetheless, these tools are paramount to improve knowledge on environmental risk assessment (ERA) associated with emerging contaminants, even when set at concentrations ≤ Environmental Quality Standards (EQS), and thus to sustainably manage natural systems under a climate change context [23].

The aim of this work was to use an ecosystem model, followed by a global sensitivity analysis (GSA), to simulate and disentangle the single and combined effects of four ECs (Bisphenol A—BPA; 4-Nonylphenol—4-NP; 17α-etinylestradiol—EE2; and Diclofenac—DCF) and temperature rise on the biomass production of four key-species from the benthic macroinvertebrate communities of an Atlantic temperate estuary (Minho estuary, NW coast of Portugal).

EC concentrations used in the simulated scenarios were the annual average Environmental Quality Standards (AA-EQS) according to the EU Directive 2013/39/EU [24]. Temperature rise scenarios were set accordingly to the moderate RCP4.5 scenario and the business-as-usual RCP8.5 scenario [25]. Subsequently, GSA [26,27] was performed to disentangle stressors’ interactions, prioritize stressor effects on biota and detect species sensitivity to the studied stressors.

We hypothesized that single ECs and their mixture at AA-EQS concentrations would not have a negative effect on macroinvertebrates, whereas the combination of ECs with temperature rise would negatively affect macroinvertebrates due to temperature-induced alterations in macroinvertebrate physiological rates and contaminant toxicity parameters.

## 2. Materials and Methods

### 2.1. Model Development

Due to the ability of combining environmental fate and the effects of conventional pollutants (e.g., nutrients) with those of toxic chemicals in aquatic systems, while describing food web dynamics, the ecological risk assessment model AQUATOX (Release 3.2) [28,29] was used to implement the Minho estuary model in a set of iterative steps [22].

**Figure 1:**
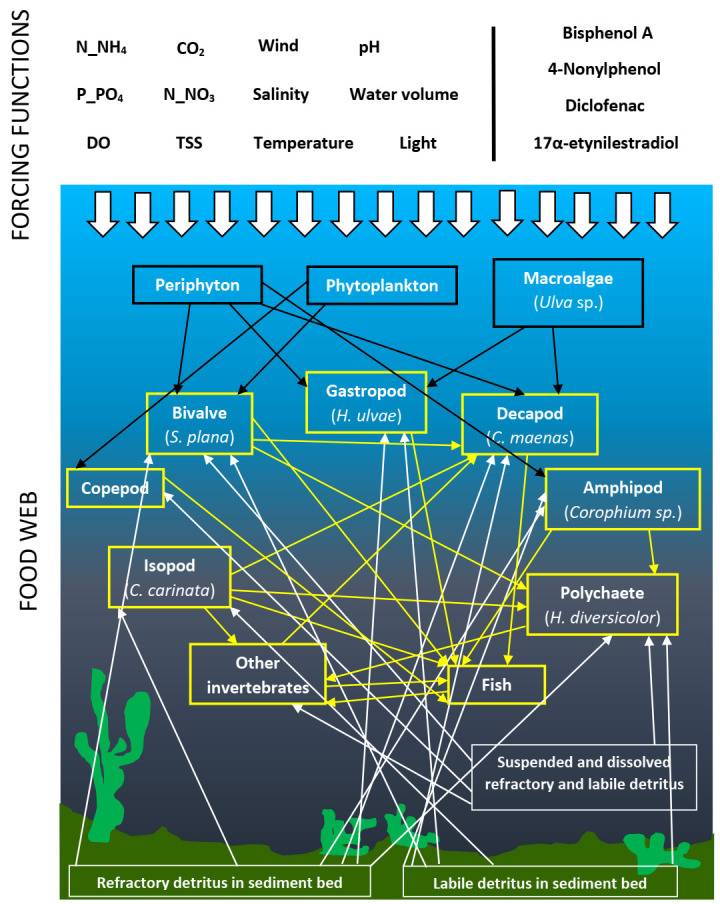
Simplified conceptual diagram of the Minho estuary model (NW coast of Portugal) used to check the effects of temperature rise, Bisphenol-A, 4-Nonlyphenol, Diclofenac and 17α-etynilestradiol on macroinvertebrates communities’ composition and production. N-NH_4_—Ammonia; N-NO_3_—Nitrate; P-PO_4_—Phosphate; DO—Dissolved Oxygen; CO_2_—Carbon Dioxide; and TSS—Total Suspended Solids. Black frames and arrows refer to primary producers. Yellow frames and arrows refer to consumers. White frames and arrows refer to sediment compartments. Upper larger white arrows refer to the effects of forcing functions in the system. The contributions of trophic groups to sediment compartments are not shown.

Briefly, the model accounts for a southern Europe estuary (Minho estuary, NW coast of Portugal: 41°53′84″ N, 08°50′44″ W) with a focus on the benthic macroinvertebrate food web, which is dominated by the clam *Scrobicularia plana*, followed by the polychaete *Hediste diversicolor*, the gastropod *Hydrobia ulvae* and the crab *Carcinus maenas*. The isopod *Cyathura carinata* and the amphipod *Corophium* sp. are also present on the sediments from the Minho estuary but in lower densities compared to the former species [30]. Primary producers (phytoplankton, periphyton and macroalgae), copepods and fish are also components of the Minho estuarine food web. According to AQUATOX, detritus were accounted by refractory and labile detritus in sediment bed, and refractory and labile suspended and dissolved detritus (Figure 1).

Information regarding other trophic groups can be found in [22,30]. Monthly values of dissolved oxygen, salinity, temperature and pH were available for the study site at the same sampling dates as biotic data [30]. Dissolved nutrients assumed constant loading values based on samples collected at the downstream area of the Minho estuary [31]. Average values of light (625 Ly d^−1^) and wind (2.2 ms^−1^) were according to data obtained at the study site (see [22]). The upstream inflow of water (185.75 m^3^s^−1^) was set to the average value measured at the Minho River during the sampling period (obtained from Confederacion Hidrográfica del Miño-Sil—HMS).

Model calibration was conducted until a satisfying fitting level between the set of observed data and simulated values was obtained, which was evaluated by the root mean square error (RMSE) estimation. After testing the model for long-term stability and sensitivity analysis, the Minho ecosystem model was considered valid to perform scenario simulations (see [22] for details).

In the present work, we will focus on the predicted biomass variations of *S. plana*, *H. diversicolor*, *H. ulvae* and *C. maenas*. In addition to being key-species from the macrofaunal communities of the Minho estuary [30], these species are also widely distributed on the mudflats of other NE Atlantic estuaries, where they can attain high densities [32,33,34,35] and play important roles in the energy transfer from lower trophic levels (microphytobenthos, detritus) to top predators (birds and fishes) [33,36,37,38].

### 2.2. ECs Brief Description and Selection

Due to its persistent and increasing use, the emerging and priority endocrine disrupting chemicals BPA, 4-NP, EE2 [39,40,41] and the pharmaceutical DCF [24] were included in the model.

BPA is a synthetic phenol extensively used in food packaging materials, dental sealants, medical devices and other human-consumption products [42]. Consequently, exposure to BPA can be ubiquitous via ingestion, inhalation and dermal contact. In the last decades, numerous studies have linked BPA to diseases such as cancer, diabetes, obesity and various disorders in the reproductive, neuronal, immune and cardiovascular systems [43,44,45]. Nowadays, manufacturers are abandoning BPA-based consumer plastics to use several “BPA-free” alternatives, such as Bisphenol S and Bisphenol P. However, BPA is still found in relatively high concentrations in rivers, lakes and estuaries worldwide [46]. Experimental evidence suggests that BPA properties (e.g., leaching) can change with temperature [42]. 4-NP, the degradation product of nonionic surfactants alkylphenol polyethoxylates, largely used as plasticizers in the manufacture of textiles, paper and agricultural chemical products, tends to accumulate in compartments with high organic content, such as sewage sludge and river sediments [47,48,49]. Reported adverse effects in aquatic organisms include impairments in the nervous system, reproduction and development processes [50,51,52]. In fact, due to their confirmed capacity to disrupt the endocrine system, NP and its ethoxylates were designated as priority hazardous substances in the Water Framework Directive and are currently under regulation [39,40]. EE2 is included in the surface water Watch List (WL) under the Water Framework Directive (WFD) for Union-wide monitoring [41]. EE2 is ranked 14th among the top 200 prescribed pharmaceuticals used as a constituent of oral contraceptives, in hormonal replacement therapy to treat menopausal and post-menopausal disorders, in the treatment of female hypogonadism, as a palliative treatment in malignant neoplasm of breast and prostate and in Turner’s syndrome [53,54]. In addition to its continuous release, EE2 is resistant to biodegradation processes due to its physical-chemical properties [55], thus posing several threats to aquatic environments. Actually, EE2 has been found to cause several disorders and modulate or disrupt developmental and reproductive processes of aquatic organisms, from algae to invertebrates, amphibians and fish [56,57,58,59,60,61]. DCF is a widely used human and veterinary pharmaceutical prescribed to reduce inflammation and control pain and is reported to be very persistent in aquatic environments [62,63]. There is a consensus that exposure to DCF can impair renal functions in vertebrates [62,63]. Although toxicity studies of DCF in invertebrates are scarce, the limited data suggest that some taxa are sensitive to DCF at low concentrations (µgL^−1^) [64,65].

### 2.3. Including ECs, LC50 and EC50 in the Minho Ecosystem Model

Given the specific parameters of a considered chemical (e.g., dissociation constant, Henry’s law constant, octanol-water partition coefficient), AQUATOX simulates the partitioning of a chemical between water, sediment and biota, accounting for the chemical’s microbial degradation, biotransformation, photolysis, hydrolysis and volatilization [29]. The ecotoxicology submodel embedded in AQUATOX simulates both lethal and sublethal acute toxic effects of chemicals in food-web organisms, as long as the user supplies the specific LC50 and EC50 values for the studied toxic chemicals. Then, a sequence of computations estimates the biomass of a given organism being lost through lethal toxicity, as well as factors that relate sublethal toxicities to the lethal toxicity [29].

In the present study, exhaustive literature reviews regarding the chemical properties of the considered ECs, the LC50 and EC50 values for the trophic groups included in the Minho food web were conducted, followed by professional scrutiny prior to incorporation in the Minho ecosystem model (Tables S1–S4). Some assumption had to be made based on the following criteria: for missing LC50 values, AQUATOX regressions were used; for missing EC50 values, it was assumed that EC10/NOEC ≈ 1 based on [66], thus, EC50 = EC10 × 4 or EC50 = NOEC × 4 and LOEC = NOEC × 2. NOEC stands for No Observed Effect Concentration and LOEC for Lowest Observed Effect Concentration.

### 2.4. Single- and Multiple-Stressors Simulated Scenarios

A total of 17 scenarios were run for ≈10 years (4020 days) with a time step of 0.1 day. EC concentrations were set as the annual average Environmental Quality Standards (AA-EQS) according to EU Directives (Table 1), and EC inflow loadings were constant throughout the simulation period. Based on IPCC predictions, average increases of 1.8 °C and 3.7 °C are expected in the RCP4.5 and the RCP8.5 scenarios, respectively [25], which were added to the observed temperature values of the control run and designated as RCP4.5 and RCP8.5 scenarios. This information is resumed in Table 1.

### 2.5. Global Sensitivity Analysis

A Global Sensitivity Analysis (GSA) was performed to check for the combined effects of ECs and temperature rise. Contrarily to one-at-a-time (OAT) methods, where single factors are perturbed with all other factors held fixed, in GSA all factors being analyzed are changed together across the full multidimensional input space [69]. This type of approach is essential when models feature nonlinearities and interactions [70].

GSA was performed by estimating standardized regression coefficients (SRC) [25,26] associated to a design matrix of combinations of the variables ‘Contaminants’ (BPA, 4-NP, EE2, DCF, mixed ECs), ‘Species’ (*S. plana*, *H. diversicolor*, *H. ulvae* and *C. maenas*), ‘Temperature’ (RCP4.5, RCP8.5) and ‘Time’ in a fully crossed design, with biomass as the dependent variable. There were 135 time-steps for each combination of factors, from 0 to 4020 days. SRC expresses the magnitude and significance of the effect of combined parameters measured using different units, as well as the explained variance, gauging the main effects of the input parameters [27].

A general linear model was fit to these data, and a stepwise procedure (both directions) was applied in order to select which independent variables should stay in the model, according to Akaike Information Criterion (AIC). Statistical analyses were carried out using the *lm* and *step* functions in the stats R package [71]. This allowed discriminating both the magnitude and significance of the effect that each variable caused in variations of biomass of the four macroinvertebrate species.

## 3. Results

**Figure 2:**
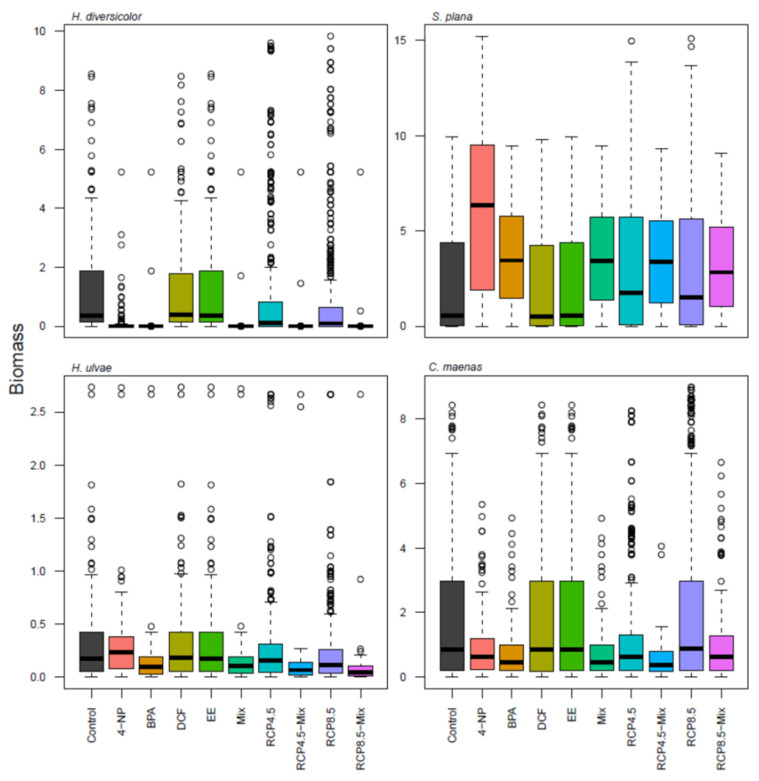
Biomass of *H. diversicolor*, *S. plana*, *H. ulvae* and *C. maenas* (gDWm^−2^) in the control run and in the simulated scenarios. Each boxplot representation is comprised by the quartiles (25 and 75%) displayed as a box, the median displayed as a horizontal black bar, standard deviation displayed as the vertical whiskers and the black dots that represent the outlier values. Mix refers to the four mixed ECs scenarios. Single EC combined with temperature scenarios are only shown in the Supplementary Materials (Figure S1).

Compared to the control run, the 10-year scenarios simulations predicted that with the exception of *S. plana*, the other three macroinvertebrates exhibit decreased biomass in the BPA, 4-NP and Mixed ECs scenarios (Figure 2). *H. diversicolor* was the most affected species, followed by *H. ulvae*, while *S. plana* increased biomass in the 4-NP, BPA and Mixed ECs scenarios. *C. maenas* was clearly enhanced in the single RCP8.5 scenario compared to the single RCP4.5 scenario. None of the four benthic macroinvertebrates was expressively affected by DCF or EE2 scenarios (Figure 2).

Despite the different responses of the four macroinvertebrate species to the tested scenarios, there was a global decreasing tendency on benthic invertebrate biomass in the mixed stressors’ scenarios (Table 2).

According to SRC analysis, ‘Species’ was the most important variable, followed by ‘Contaminant’ and ‘Time’. All levels of these variables had highly significant coefficients, except for *S. plana*, which was not significantly different from *C. maenas*, the reference level for ‘Species’ (Table 3). *H. diversicolor* was the most affected species, followed by *H. ulvae*. As to the contaminants, compared to 4-NP (the reference level for ‘Contaminant’), BPA and the four mixed ECs caused the highest decreases on the biomass of macroinvertebrates.

Despite the differences in the importance of the variables, all of them were necessary for attaining the significant lower AIC value in the model (Table 4).

## 4. Discussion

Our initial hypotheses were not confirmed since BPA and the mixed ECs scenarios had the most adverse effects on the biomass production of the macroinvertebrates from the Minho estuary, which exhibited a general decreasing tendency over time. However, different species presented different sensitivity to the simulated scenarios, which in turn induced disturbances, namely, trophic cascade effects across the food web. *H. diversicolor* was the most sensitive species to 4-NP, BPA and mixed ECs, and *C. maenas* was quite sensitive to 4-NP and BPA. On the other hand, the polychaete and the crab have omnivorous feeding habits that include predation on *S. plana* [72,73], which is accounted for in the model. Thus, the decreased predation by both the polychaete and the crab on *S. plana* led to an increase in the biomass of the clam in the 4-NP, BPA and mixed ECs scenarios. In line with this, it has been shown that disturbances in trophic levels may lead to trophic cascades, which can affect and control entire marine ecosystems [74].

Moreover, coupling numerical models with experimental approaches that assess the effects of mixtures of contaminants at multiple levels of biological organization [11] may be extremely useful to understand and simulate the potential impacts of contaminants, including ECs, at the sub-organism, organism, community and ecosystem levels. In turn, such multi-level approaches followed by numerical projections of impacts over time will potentially meet the criteria of newer concepts of biodiversity conservation that encompass assessing toxicant effects on molecules, cells, organisms and communities [11].

GSA was paramount to highlight the differences regarding ECs impacts and species’ sensitivity. Thus, we recommend that complex end-to-end models of marine ecosystems account for global sensitivity analysis as a way to filter the complexity of results linked to ecosystem models and, therefore, prioritize stressors, highlight stressor–trophic groups’ interactions and detect cumulative effects. This type of approach is of significant importance to strengthen communication and understanding between modelers and environmental managers and thus guide decision-making. Indeed, sensitivity analysis is prescribed in national and international guidelines in the context of impact assessment [70].

In addition to accounting for the physical and biogeochemical characteristics of the study site, the Minho ecosystem model also simulates the transformations of ECs in the water and sediment and the acute lethal and non-lethal toxic effects on biota [29,75]. Additionally, because the model also describes trophic relationships among biotic groups, it can simulate the indirect effects of contaminants and other stressors throughout the food web [76,77]. Models such as the present one are important research tools due to the strong evidence for nonlinear interactions among stressors acting on aquatic and coastal systems [17,18,78,79].

Despite acknowledging that the Minho ecosystem model can be improved by adding up more data and further calibration effort, single-scenario predictions are globally in agreement with extensive literature data, which report no significant ecotoxicological effects of DCF at environmentally relevant concentration to aquatic organisms [65]. EE2 can be accumulated by aquatic animals from different exposure routes, which may lead to higher internal levels of EE2 than the ones usually quantified by experimental approaches focusing on a single exposure route [54]. This may be relevant and would require further research due to the extensive use of EE2 as an oral contraceptive and in hormone replacement therapy applied to post-menopausal disorders [54]. BPA and 4-NP are recognized as ubiquitous and environmentally persistent [80], with reports of adverse effects of BPA on aquatic organisms [81] and of high environmental risk quotients of 4-NP in coastal waters [82].

In practice, the present methodology can be used to prioritize down-the-drain contaminants, setting EQS ranges for worldwide abundant ECs mixtures and assessing the most sensitive trophic groups that could then be used as bioindicators in a specific system. Based on the present results, we recommend a revision of the EQS values of BPA and 4-NP, and the use of *H. diversicolor* and *S. plana* as bioindicator species in the Minho estuary.

Furthermore, complementing the present approach with the use of ecotoxicological biomarkers in key-species [2] may provide important information that allows the classification of low-impacted estuaries, such as the Minho river–estuary system, within the Water Framework Directive 2000/60/EC (WFD) context [2].

Globally, the tested temperature rise scenarios had no significant effect on the biomass of the benthic macroinvertebrates from the Minho estuary, although the business-as-usual scenario (RCP8.5 av. increase = 3.7 °C) was close to having a significant effect on the biomass variation of the studied species (*p* = 0.07). This is in agreement with projections indicating that climate-related risks depend on the rate, peak and duration of the warming, which will be larger if global warming exceeds 1.5 °C [83]. Interestingly, according to simulations, *C. maenas* was able to cope with the temperature increase induced by the RCP8.5 scenario, corroborating the high physiological plasticity of the crab [84], which can increase predation and feeding rates at warmer temperatures [85].

The AQUATOX model of the Minho estuary accounts for site-specific characteristics, such as the system volume, surface area and mean depth; however, a higher spatial resolution of the system site can be achieved by, e.g., adding a multi-layer sediment model, creating a multi-segment model or setting up complex linkages [28]. Nevertheless, this would require more data and a significant increase in the number of hours devoted to model calibration and verification, which was not the goal of the present study, although it can be undertaken in the future.

Previous works have presented coupled process-based modeling and empirical models [86], random forest models [87], probabilistic ERA frameworks with prevalence plots [88] and others [89] to tackle the impact of multiple stressors in aquatic systems. The present work adds up to this by introducing an approach that, in addition to prioritizing stressors, also highlights the most sensitive trophic groups to the tested stressors and provides clues on how direct and indirect effects transfer across the food web. Although site- and species-specific calibrations are required, the present methodology has the potential to be applicable to other ecosystems and effectively support the management of estuaries and coastal areas.

## 5. Conclusions

Our results support the idea that a methodology combining AQUATOX ecosystem models with GSA based on SRC estimation is useful to quantify the effects of stressors on biotic responses, prioritize stressors, identify the most sensitive trophic groups and recognize cascade effects across food webs. This type of approach is important to support researchers, resource managers and policy makers on developing strategies to reduce or reverse the ecological, economic and social impacts of environmental stressors [16]. Notwithstanding, the application of the present methodology within a coastal management perspective requires thorough site- and species-specific model calibration, sensitivity analysis and uncertainty estimation.

Ecological models of coastal systems, such as the present one, may also reveal useful to support theoretical modelling approaches related to the responses of trophic groups to temperature, salinity and other climate-related variables, and thus provide guidance for field-scale research linked to climate change effects and interactions on aquatic food webs.

Furthermore, we recommend that food web models of rivers and estuaries be used as tools to support implementation of legislation regarding the Environmental Quality Standards (EQS) of down-the-drain contaminants, particularly the ones widely used by human populations.

## Figures and Tables

**Table 1:** Details of the simulated scenarios: Scenario name, contaminant added to simulation, contaminant concentration during simulation, temperature increase added to simulation, notes and literature references. RCP—Relative Concentration Pathway (temperature increase); AA-EQS—annual average Environmental Quality Standards concentration. - Not Applicable.

Scenario Name	Contaminant	Concentration (µgL^−1^)	Temperature Increase (°C)	Note	Reference
RCP4.5	Without any EC	-	+1.8	-	[25]
RCP8.5	Without any EC	-	+3.7	-	[25]
4-NP	4-Nonylphenol	0.3	-	AA-EQS	[39,40]
4-NP-RCP4.5	4-Nonylphenol	0.3	+1.8	AA-EQS	[39,40]
4-NP-RCP8.5	4-Nonylphenol	0.3	+3.7	AA-EQS	[39,40]
BPA	Bisphenol A	0.2	-	AA-EQS	[24]
BPA-RCP4.5	Bisphenol A	0.2	+1.8	AA-EQS	[24]
BPA-RCP8.5	Bisphenol A	0.2	+3.7	AA-EQS	[24]
DCF	Diclofenac	0.1	-	AA-EQS	[67,68]
DCF-RCP4.5	Diclofenac	0.1	+1.8	AA-EQS	[67,68]
DCF-RCP8.5	Diclofenac	0.1	+3.7	AA-EQS	[67,68]
EE_2_	17α-ethinylestradiol	3.5 × 10^−5^	-	AA-EQS	[24,68]
EE_2_-RCP4.5	17α-ethinylestradiol	3.5 × 10^−5^	+1.8	AA-EQS	[24,68]
EE_2_-RCP8.5	17α-ethinylestradiol	3.5 × 10^−5^	+3.7	AA-EQS	[24,68]
Multi-EC	4-NP, BPA, DCF, EE_2_	The same as above	-	AA-EQS	The same as above
Multi-EC-RCP4.5	4-NP, BPA, DCF, EE_2_	The same as above	+1.8	AA-EQS	The same as above
Multi-EC-RCP8.5	4-NP, BPA, DCF, EE_2_	The same as above	+3.7	AA-EQS	The same as above

**Table 2:** Benthic invertebrate biomass production in the Minho estuary during the control run, single scenarios and four of the simulated mixed stressor scenarios (scenarios of single EC combined with temperature increases are not shown).

Biomass Production	Control	BPA	4NP	EE_2_	DCF	RCP4.5	RCP8.5	Mixed ECs	Mixed ECs-RCP4.5	Mixed ECs-RCP8.5
Benthic Invertebrates (gDWm^−2^)	15.39	14.99	17.62	16.20	16.15	14.84	15.34	14.92	14.46	14.45

**Table 3:** General linear model coefficients and their significance for the variables employed in the SRC.

Variable	Estimate	Std. Error	*t* Value	Pr (>|t|)
BPA	−2.72	0.15	−18.35	<0.001
Control	1.51	0.15	10.15	<0.001
DCF	1.50	0.15	10.02	<0.001
EE2	1.51	0.15	10.12	<0.001
Mixed EC	−2.67	0.15	−17.97	<0.001
*H. diversicolor*	−7.08	0.12	−58.40	<0.001
*H. ulvae*	−1.93	0.12	−15.90	<0.001
*S. plana*	0.62	0.12	5.08	0.335
Time	−3 × 10^−4^	4 × 10^−5^	−8.70	<0.001

**Table 4:** AIC values of the models fit in the stepwise method.

Variable Removed	*df*	Sum of Squares	Residual Sum of Squares	*AIC*
None			173,404	28,027.59
Temperature	2	58	173,346	28,028.34
Time	1	1347	174,751	28,101
Contaminant	5	34,324	207,728	29,773
Species	3	88,959	262,363	32,047

## Data Availability

Not applicable.

## Supplementary Materials

The following supporting information can be downloaded at: https://www.mdpi.com/article/10.3390/toxics10020046/s1, Figure S1: Biomass of the four macroinvertebrate species (gDWm-2) in single EC coupled with temperature rise scenarios, Table S1: BPA ecotoxicological parameters, Table S2: DCF ecotoxicological parameters, Table S3: EE2 ecotoxicological parameters, Table S4: 4-NP ecotoxicological parameters.
