# Supplementary material for: Prioritizing the Effects of Emerging Contaminants on Estuarine Production under Global Warming Scenarios
Source: Toxics. 2022 Jan 20;10(2):46. doi: 10.3390/toxics10020046 (PMC8877751; doi:10.3390/toxics10020046)
Supplement: Supplementary file 1 [file toxics-10-00046-s001.zip › toxics-1499713-supplementary.pdf]

toxics

Supplementary Materials: Prioritizing the Effects of Emerging Contaminants on Estuarine Production under Global Warming Scenarios

Irene Martins, Joana Soares, Teresa Neuparth, Aldo F. Barreiro, Cândido Xavier, Carlos Antunes and Miguel M. Santos

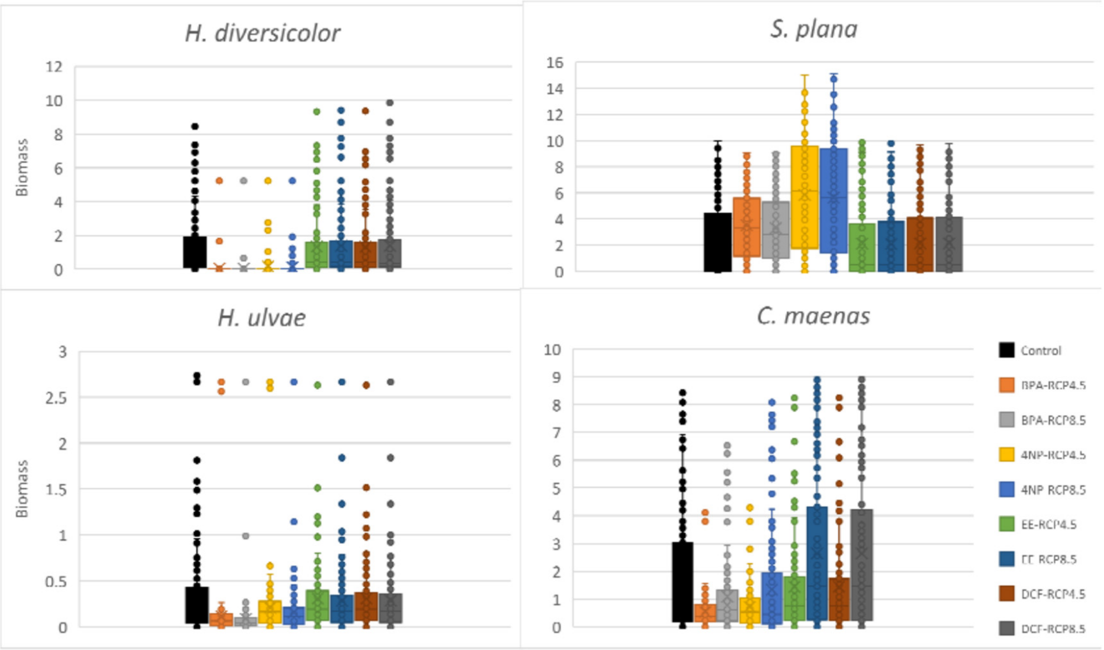

Figure S1. Biomass of the four macroinvertebrate species (gDWm^{-2}) in single EC coupled with temperature rise scenarios. Each boxplot representation is comprised by the quartiles (25 and 75%) displayed as a box, the median displayed as a horizontal black bar, standard deviation displayed as the vertical whiskers, and the black dots that represent the outlier values.

Table S1. BPA ecotoxicological parameters.

Functional Group	LC_{50} (μgL^{-1})	LC_{50} Exp. Time (h)	K2 elim. Rate Const (1/d)	$EC_{50growth}$ (μgL^{-1})	Growth Exp. Time (h)	$EC_{50reprod}$ (μgL^{-1})	Reprod. Exp. time (h)	Source
Amphipod/Isopod/Decapod	5600	48	0.014	2560	504	2560	504	[1,2]
Clam	7043.9	96	0.014	50	504	50	504	AQUATOX Regression; [3]
Copepod	4320	48	0.014	550	96	550	96	[4,5]
Gastropod	11100	96	0.014	1.02	96	1.02	96	[6]
Polychaete	227	96	0.014	720	72	720	72	[7]
Fish	9400	96	1	14560	672	3.5	2400	[3,8]

Plant Functional Group	$EC50_{photo}$ (μgL^{-1})	EC50 $_{exp. time}$ (h)	K2 elim. Rate const (1/d)	LC50 (μgL^{-1})	LC50 exp. Time (h)	Source
Greens	1100	96	3.036	1100	96	[8]
Diatoms	1100	96	3.036	1100	96	[8]
Bluegreens	1100	96	3.036	1100	96	[8]
Macrophytes	31200	168	0.158	31200	168	[9]

Table S2. DCF ecotoxicological parameters.

Animal Functional Group	LC_{50} (μgL⁻¹)	LC_{50} exp. Time (h)	K2 elim. Rate Const (1/d)	$EC_{50growth}$ (μgL⁻¹)	Growth Exp. Time (h)	$EC_{50reprod}$ (μgL⁻¹)	Reprod. Exp. Time (h)	Source
Amphipod/Isopod/Decapod	10436	48	0.014	16300	96	28100	504	[10,11]
Clam	5207.775	504	0.014	16300	96	28100	504	AQUATOX regression; [11]
Copepod	15800	48	0.014	16300	96	28100	504	[10,11]
Gastropod	1075.74	504	0.014	16300	96	28100	504	AQUATOX regression; [11]
Polychaete	6397.972	504	0.014	16300	96	28100	504	AQUATOX regression; [11]
Fish	10100	96	1	4524	504	4524	504	[11,12]

Plant Functional Group	$EC50_{photo}$ (μgL⁻¹)	EC50 exp. time (h)	K2 elim. Rate Const (1/d)	LC50 (μgL⁻¹)	LC50 Exp. Time (h)	Source
Greens	185600	96	3.036	185600	96	[10]
Diatoms	185600	96	3.036	185600	96	[10]
Bluegreens	185600	96	3.036	185600	96	[10]
Macrophytes	47600	168	0.158	47600	168	[10]

Table S3. EE_2 ecotoxicological parameters.

Animal Functional Group	LC_{50} (μgL⁻¹)	LC_{50} Exp. Time (h)	K2 elim. Rate Const (1/d)	$EC_{50growth}$ (μgL⁻¹)	Growth Exp. Time (h)	$EC_{50reprod}$ (μgL−1)	Reprod. Exp. Time (h)	Source
Amphipod/Isopod/ Decapod	614.507	48	0.014	2000	504	2000	504	AQUATOX regression; [13]
Clam	1621.507	48	0.014	2000	504	2000	504	AQUATOX regression; [13]
Copepod	1100	48	0.014	184	120	184	120	[14]
Gastropod	100.325	48	0.014	2000	504	2000	504	AQUATOX regression; [13]
Polychaete	1442.137	48	0.014	2000	504	2000	504	AQUATOX regression; [13]
Fish	1700	96	1	0.024	5400	0.002	5040	[15,16]

Plant Functional Group	$EC50_{photo}$ (μgL¹)	EC50 exp. time (h)	K2 elim. Rate Const (1/d)	LC50 (μgL⁻¹)	LC50 Exp. Time (h)	Sources
Greens	1013	96	3.036	1013	96	AQUATOX regression
Diatoms	216	72	3.036	216	72	[17]
Bluegreens	1013	96	3.036	1013	96	AQUATOX regression
Macrophytes	3000	96	0.158	3000	96	[18,19]

Table S4. 4-NP ecotoxicological parameters.

Animal Functional Group	LC50 (μgL⁻¹)	LC50 exp. Time (h)	K2 elim. Rate Const (1/d)	$EC50_{growth}$ (μgL⁻¹)	Growth Exp. Time (h)	$EC50_{reprod}$ (μgL⁻¹)	Reprod. Exp. time (h)	Source
Amphipod/Isopod/ Decapod	62	96	0.014	20.7	96	20.7	96	[18–20]
Clam	38	96	0.014	19	96	19	96	[20]
Copepod	276	96	0.014	69	96	69	96	[21]
Gastropod	774	96	0.014	378	96	378	96	[18,19]

Polychaete	342	96	0.014	268	96	268	96	[18,19]
Fish	128	96	1	96	96	96	96	[18,19]

**Plant Functional Group**	**EC50$_{photo}$ (µgL^{-1})**	**EC50 $_{exp. time}$ (h)**	**K2 elim. Rate Const (1/d)**	**LC50 (µgL^{-1})**	**LC50 exp. Time (h)**	**Source**
Greens	410	96	3.036	410	96	[22–25]
Diatoms	410	96	3.036	410	96	[22–25]
Bluegreens	410	96	3.036	410	96	[22–25]
Macrophytes	3000	96	0.158	3000	96	[18,19]

References

1. Watts, M.M.; Pascoe, D.; Carroll, K. Survival and precopulatory behaviour of *Gammarus pulex* (L.) exposed to two xenoestrogens. *Water Res.* **2001**, *35*, 2347–2352
2. Plahuta, M.; Tišler, T.; Pintar, A.; Toman, M.J. Adverse effects of bisphenol A on water louse (*Asellus aquaticus*). Ecotoxicol. Environ. Saf. 2015, 117, 81–88, https://doi.org/10.1016/j.ecoenv.2015.03.031.
3. Flint, S.; Markle, T.; Thompson, S.; Wallace, E. Bisphenol A exposure, effects, and policy: A wildlife perspective. J. Environ. Manag. 2012, 104, 19–34, https://doi.org/10.1016/j.jenvman.2012.03.021.
4. Marcial, H.S.; Hagiwara, A.; Snell, T.W. Estrogenic compounds affect development of harpacticoid copepod Tigriopus japonicus. Environ. Toxicol. Chem. 2003, 22, 3025–3030.
5. Andersen, H.R.; Halling–Sørensen, B.; Kusk, K.O. A parameter for detecting estrogenic exposure in the copepod Acar–tia tonsa. *Ecotoxicol. Environ. Safe*. **1999**, *44*, 56–61.
6. Andrade, A.L.C.; Soares, P.R.L.; Silva, S.C.B.L.; Silva, M.C.G.; Santos, T.P.; Cadena, M.R.S.; Soares, P.C.; Cadena, P.G. Evaluation of the toxic effect of endocrine disruptor Bisphenol A (BPA) in the acute and chronic toxicity tests with *Pomacea lineata* gastropod. *Comp. Biochem. Physiol. Part C* **2017**, *197*, 1–7, http://dx.doi.org/10.1016/j.cbpc.2017.04.002.
7. Roepke, T.A.; Snyder, M.J.; Cherr, G.N. Estradiol and endocrine disrupting compounds adversely affect development of sea urchin embryos at environmentally relevant concentrations. Aquat. Toxicol. 2005, 71, 155–173, https://doi.org/10.1016/j.aquatox.2004.11.003
8. Alexander, H.C., Dill, D.C., Smith, L.W., Guiney, P.D., Dorn, P. (1988). Bisphenol–A: Acute aquatic toxicity. Environ. Toxicol. Chem. 7, 19–26.
9. Putt, A.E. Bisphenol A—7–Day Toxicity to Duckweed, *Lemna Gibba*, under Static–Renewal Conditions; Springborn Smithers Laboratories, Inc.: Wareham, MA, USA, 2003.
10. ECOTOX. Available online: https://cfpub.epa.gov/ecotox/(accessed several times during 2020 and 2021).
11. Ferrari, B.; Mons, R.; Vollat, B.; Fraysse, B.; Garric, J. Environmental Risk Assessment of Six Human Pharmaceuticals: Are the current environmental risk assessment procedures sufficient for the protection of the aquatic environment? *Environ. Tox. Chem.* **2004**, *23*, 1344–1354.
12. Praskova, E.; Voslarova, E.; Sikorova, Z.; Plhalova, L.; Svobodova, Z. Assessment of diclofenac LC50 reference val–ues in juvenile and embryonic stages of the zebrafish (*Danio rerio*). *Polish J. Vet. Sc.* **2011**, *14*, 545–549.
13. Clubbs, R.L.; Brooks, B.W. *Daphnia magna* responses to a vertebrate estrogen receptor agonist and an antagonist: A multigenerational study. *Ecotoxicol. Environ. Saf.* **2007**, *67*, 385–398, https://doi.org/10.1016/j.ecoenv.2007.01.009.
14. Anderson, P.D.; D'Aco, V.J.; Shanahan, P.; Chapra, S.C.; Buzby, M.E.; Cunningham, V.L.; DuPlessie, B.M.; Hayes, E.P.; Mastrocco, F.J.; Parke, N.J.; et al. Screening Analysis of Human Pharmaceutical Compounds in U.S. Surface Waters. *Environ. Sci. Technol.* **2003**, *38*, 838–849.
15. Schäfers, C.; Teigeler, M.; Wenzel, A.; Maack, G.; Fenske, M.; Segner, H. Concentration– and Time–dependent Effects of the Synthetic Estrogen, 17α–ethinylestradiol, on Reproductive Capabilities of the Zebrafish, *Danio rerio*. *J. Toxicol. Environ. Health Part A* **2007**, *70*, 768–779.
16. Nash, J.P.; Kime, D.E.; Van der Ven, L.T.; Wester, P.W.; Brion, F.; Maack, G.; Stahlschmidt– Allner, P.; Tyler, C.R. Long–Term Exposure to Environmental Concentrations of the Pharmaceutical Ethynylestradiol Causes Reproductive Failure in Fish. *Environ. Health Perspect.* **2004**, *112*, 1725–1733.
17. Kopf, W. Wirkung endokriner SToffe in Biotests mit Wasserorganismen. Vortag bei der 50. Factagung des BAy. In *LA fur Wasserwritschaft: Stoffe mit endokriner Wirkung im Wasser (abstract) in German*; **1995**; pp. 82–100.
18. Brooke, L.T. Acute and Chronic Toxicity of Nonylphenol to Ten Species of Aquatic Organisms. US–EPA Contract No 68–C1–0034, 1–36. Duluth, MN, US Environmental Protection Agency, 1**993**

19. Brooke, L.T. Accumulation and Lethality for Two Freshwater Fishes (Fathead Minnow and Bluegill) to Nonylphenol. US–EPA Contract No 68–C1–0034; Duluth, MN, US Environmental Protection Agency **1993.**
20. Lussier, S.; Champlin, D.; LiVolsi, J.; Poucher, S.; Pruell, R.; Thursby, G. Acute Toxicity of 4–Nonylphenol to Saltwater Animals. US–PEA Draft Report, **1997.**
21. England, D.E. Chronic Toxicity of Nonylphenol to Ceriodaphnia Dubia. Analytical Biochemistry Laboratories, Inc. Report No. 41509. Washington D.C., Chemical Manufacturers Association, 159 pp. **1995.**
22. Ward, T.J.; Boeri, R.L. *Acute Static Toxicity of Nonylphenol to the Marine Alga Skeletonema Costatum*. Report No. 8970–CMA. In: Four Environmental Effects 4–Nonylphenol final reports. Envirosystems Division, Resource Analysts Inc., Washington D.C., Alkylphenol & Ethoxylates Panel, Chemical Manufacturers Association. 42 pp. **1990**
23. Ward, T.J.; Boeri, R.L. *Acute Static Toxicity of Nonylphenol to the Freshwater Alga (Selenastrum Capricornutum)*; Report No. 8969–CMA. In: Four Environmental Effects 4–Nonylphenol final reports. Envirosystems Division, Resource Analysts Inc., Washington D.C., Alkylphenol & Ethoxylates Panel, Chemical Manufacturers Association. 41 pp. **1990**
24. Ward, T.J.; Boeri, R.L. *Acute Flow through Toxicity of Nonylphenol to the Mysid (Mysidopsis Bahia);* Report No. 8974–CMA. In: Four Environmental Effects 4–Nonylphenol final reports. Envirosystems Division, Resource Analysts Inc., Washington D.C., Alkylphenol & Ethoxylates Panel, Chemical Manufacturers Association. 35 pp. **1990**
25. Ward, T.J.; Boeri, R.L. *Acute Flow through Toxicity of Nonylphenol to the Sheepshead Minnow (Cyprinodon Varie–Gatus);* Report No. 8972–CMA. In: Four Environmental Effects 4–Nonylphenol final reports. Envirosystems Division, Resource Analysts Inc., Washington D.C., Alkylphenol & Ethoxylates Panel, Chemical Manufacturers Association. 34 pp. **1990.**
